# Enhanced Optoelectronic Properties of PFO/Fluorol 7GA Hybrid Light Emitting Diodes via Additions of TiO_2_ Nanoparticles

**DOI:** 10.3390/polym8090334

**Published:** 2016-09-06

**Authors:** Bandar Ali Al-Asbahi, Mohammad Hafizuddin Haji Jumali, Mohamad Saleh AlSalhi

**Affiliations:** 1 Department of Physics and Astronomy, Collage of Science, King Saud University, Riyadh 11451, Saudi Arabia; malsalhy@gmail.com; 2 Department of Physics, Faculty of Science, Sana’a University, PO Box 12544, Sana’a, Yemen; 3 School of Applied Physics, Faculty of Science and Technology, Universiti Kebangsaan Malaysia, UKM Bangi, Selangor 43600, Malaysia; hafizhj@ukm.edu.my; 4 Research Chair in Laser Diagnosis of Cancers, Collage of Science, King Saud University, Riyadh 11451, Saudi Arabia

**Keywords:** poly(9,9′-di-*n*-octylfluorenyl-2,7-diyl) (PFO)/Fluorol 7GA blend, organic light emitting diode (OLED), electroluminescence, turn-on voltage, luminance efficiency

## Abstract

The effect of TiO_2_ nanoparticle (NP) content on the improvement of poly(9,9′-di-*n*-octylfluorenyl-2,7-diyl) (PFO)/Fluorol 7GA organic light emitting diode (OLED) performance is demonstrated here. The PFO/Fluorol 7GA blend with specific ratios of TiO_2_ NPs was prepared via a solution blending method before being spin-coated onto an indium tin oxide (ITO) substrate to act as an emissive layer in OLEDs. A thin aluminum layer as top electrode was deposited onto the emissive layer using the electron beam chamber. Improvement electron injection from the cathode was achieved upon incorporation of TiO_2_ NPs into the PFO/Fluorol 7GA blend, thus producing devices with intense luminance and lower turn-on voltage. The ITO/(PFO/Fluorol 7GA/TiO_2_)/Al OLED device exhibited maximum electroluminescence intensity and luminance at 25 wt % of TiO_2_ NPs, while maximum luminance efficiency was achieved with 15 wt % TiO_2_ NP content. In addition, this work proved that the performance of the devices was strongly affected by the surface morphology, which in turn depended on the TiO_2_ NP content.

## 1. Introduction

Despite recent advancement, conjugated polymers, which are well known as active materials in various photovoltaic [1,2] and electroluminescence [3,4,5] devices, continue to inherit two major problems, namely poor stability and low luminance efficiency [6,7]. The main reason for the former lies on photo-oxidation, while the latter is closely associated with low charge carrier mobility and high-energy barrier for its injection.

One way to improve the transport and charge injection limitations is via incorporation of inorganic semiconductors into conjugating polymers [8,9]. Previous works have successfully demonstrated the additions of the TiO_2_ NPs in conjugated polymers, namely the poly(9,9-di-*n*-octylfluorenyl-2,7-diyl) (PFO) reduced energy gap of PFO, increased the density of localized states within the forbidden gap, improved conformational disorder, and ultimately enhanced the organic light emitting diode (OLED) performance [10,11].

Another approach, suggested to improve the electroluminescence properties of conjugated polymer OLED, is via donor/acceptor blending [12,13]. This was clearly observed and reported in our recent work on blending an acceptor, namely Fluorol 7GA, into PFO [14,15]. Due to compatible energy levels [16], enhancement in the luminance and reduction in turn-on voltage were recorded. However, these improvements were hindered by the formation of excimer. In addition, the donor/acceptor blending approach commonly resulted in the formation of non-luminescent fluorophores in the solid state form, even though they are highly emissive in solution. This is due to the quenching of the emission caused by intermolecular interactions energy transfer [17].

In this paper, the additions of TiO_2_ NPs into a PFO/Fluorol 7GA blend is presented as a novel method to improve the performance of the PFO-based OLED device. The effect of TiO_2_ NP content on performance of the PFO/Fluorol 7GA OLED in terms of turn-on voltage, electroluminescence spectra (EL), luminance, and luminance efficiency are reported. In addition, the effect of the film morphology on the device performance is also demonstrated.

## 2. Experimental Procedures

### 2.1. Materials

**Figure 1:**
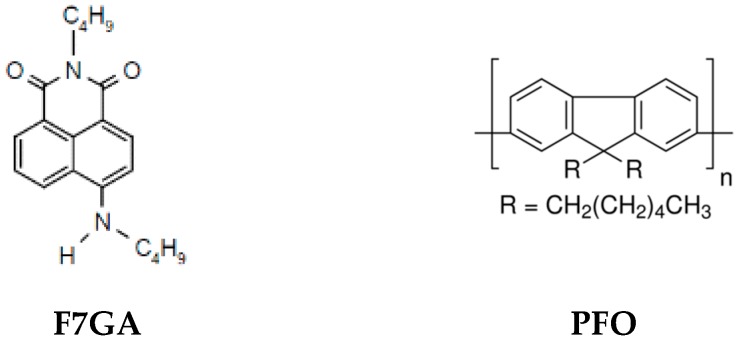
The chemical structures of poly(9,9′-di-*n*-octylfluorenyl-2,7-diyl) (PFO) and Fluorol 7GA.

Poly(9,9′-di-*n*-octylfluorenyl-2,7-diyl) (PFO) (*M*_W_ = 58,200) and TiO_2_ powder (P25, with mean size of primary particles of 25 nm) were purchased from Sigma-Aldrich (St. Louis, MO, USA), while the fluorescent dye (Fluorol 7GA) was purchased from Exciton (Dayton, Ohio, USA). These materials were stored in a dry box and used as received. All materials were dissolved in toluene solvent produced by Fluka (Buchs, Switzerland). Indium tin oxide (ITO)-coated glass with a sheet resistance of 50 Ω/sq purchased from Merck Balzers (Balzers, Liechtenstein) was used as a substrate in this study. The chemical structures of PFO and Fluorol 7GA are presented in Figure 1.

### 2.2. Samples Preparation and OLEDs Fabrication

The solution blending method was used to prepare PFO/Fluorol 7GA/TiO_2_ nanocomposites. Prior to the blending, PFO and Fluorol 7GA were dissolved separately in toluene to form stock solutions. Next, 1.0 mg/mL Flourol 7GA solution was added to 15 mg/mL PFO solution to form a blend. In this work, the Flourol 7GA content was fixed at 0.5 wt % of PFO. Then, different weight ratios of TiO_2_ NPs (5, 10, 15, 20, 25, 30, and 35 wt %) were added to the blend. The blends were stirred at 600 rpm overnight, followed by sonication for 1 h to improve the homogeneity of the nanocomposites.

The ITO coated glass that was used as an anode for fabrication of the OLED was prepared by etching a certain area using vapor of nitric acid (HNO_3_) and hydrochloric acid (HCl) in a ratio of 3:1. This step was followed by sequential cleaning in iso-propanol and acetone under ultrasonication for 10 min.

A total of 100 μL of the PFO/Fluorol 7GA/TiO_2_ blend was deposited onto the substrate with dimensions of 1.2 × 2 cm by a spin coating technique to form a film. The deposition parameters were fixed at a rotational speed of 2000 rpm for 30 s. Then, it was baked at 120 °C for 10 minutes in a vacuum oven to remove the solvent from the film. As for the fabrication of OLED, a thin aluminum layer (to serve as cathode) was deposited onto the film using the electron beam technique. The deposition rate used in this work was fixed at 2 Å/min at the chamber pressure of 2.5 × 10^−6^ Pa. The active area and thickness of the aluminum cathode prepared were 0.076 cm^2^ and 150 nm, respectively.

### 2.3. Samples Characterization

The current density–voltage (J–V), electroluminescence spectra (EL), and luminance were obtained using a Keithley 238 measurement system (Cleveland, OH, USA) and HR2000 Ocean Optic Spectrometer (Metric Drive, FL, USA). The surface morphology of the films was investigated by Scanning Probe Microscope (SPM) (Stoughton, MA, USA) in tapping mode using a phosphorous-doped Si probe (model Veeco CONT20A-CP, part No. MPP-31123-10).

## 3. Results and Discussion

### 3.1. Current Density–Voltage Measurements

**Figure 2:**
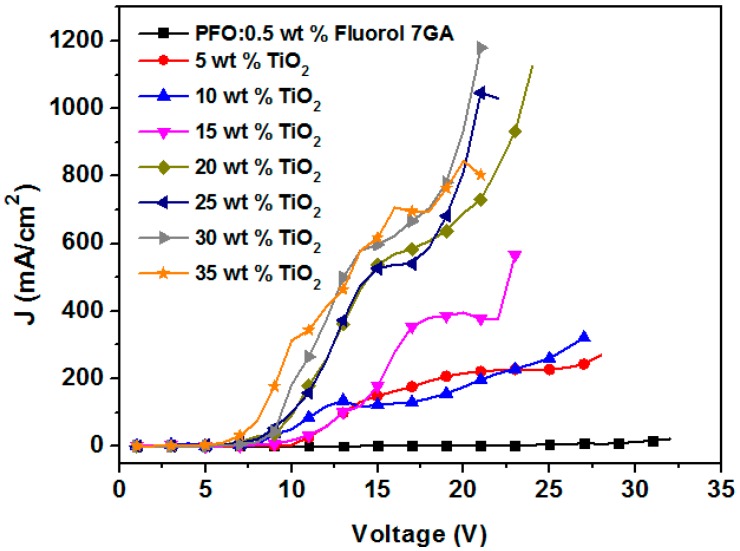
Current density–voltage (J–V) characteristics of the PFO/Fluorol 7GA/TiO_2_ organic light emitting diode (OLED) devices.

The incorporation of TiO_2_ NPs into the PFO/Fluorol 7GA blend was investigated in terms of current density–voltage (J–V) characteristics and is shown in Figure 2. It was found that the current density increased with the TiO_2_ NP content, while the turn-on voltage exhibited an opposite trend, proving the enhancement of the device’s performance (Table 1).

**Figure 3:**
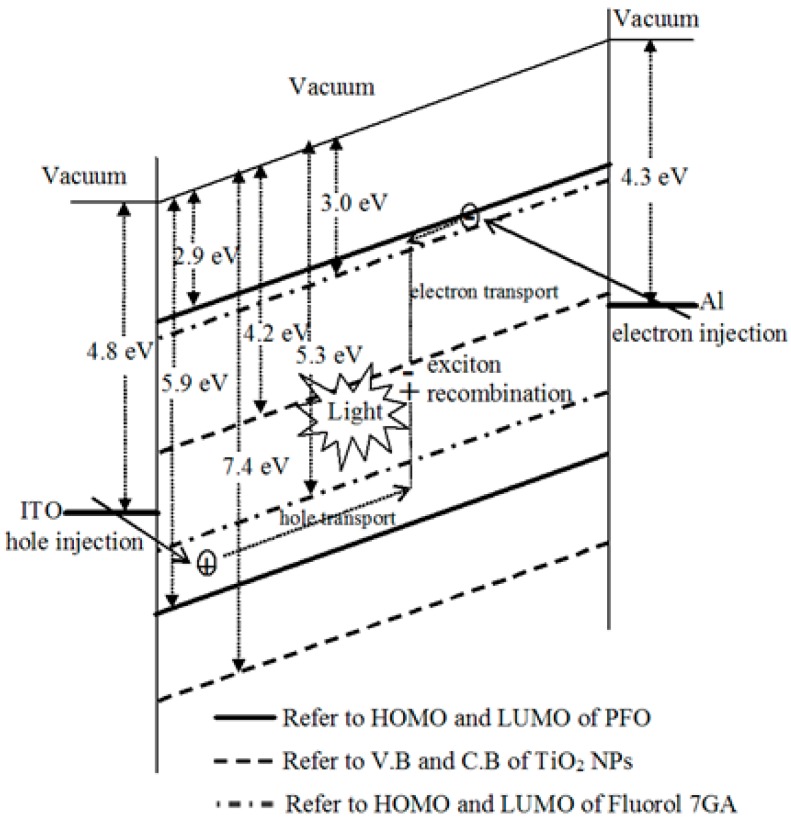
Energy band diagram for Al/PFO:Fluorol 7GA:TiO_2_/ITO OLED device relative to vacuum.

Greater current density was recorded due to a decrease in the resistance and activation energy of the emitting layer [18,19]. The turn-on voltage of the nanocomposite OLEDs reduced from 17 V in the PFO/Fluorol 7GA device to 12 V in the PFO/Flourol 7GA/TiO_2_ OLEDs. This reduction indicated an improved electron injection in the nanocomposite OLED, resulting from a lower potential barrier for charge injection. The lowest unoccupied molecular orbital (LUMO) barrier height was reduced from 1.3 eV, in the case of PFO/Fluorol 7GA, to 0.1 eV upon the addition of TiO_2_ NPs, as illustrated in Figure 3.

### 3.2. Electroluminescence Spectra

**Figure 4:**
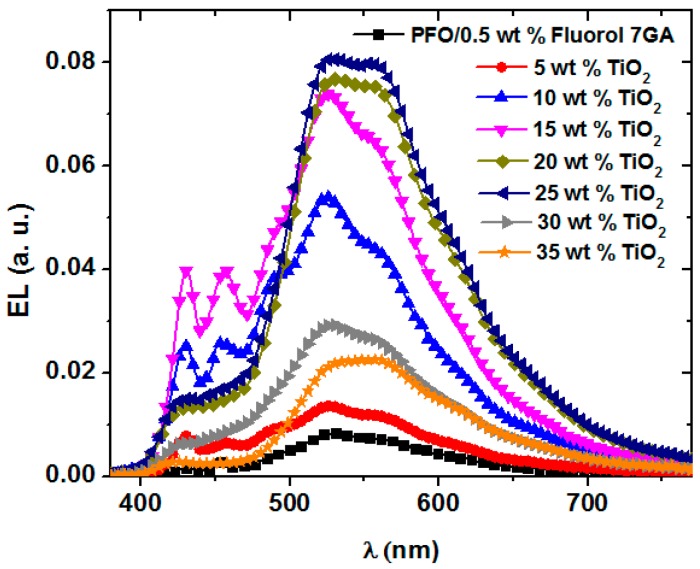
EL spectra of PFO/Fluorol 7GA/TiO_2_ devices at applied voltages corresponding to maximum luminance.

The electroluminescence (EL) spectra of PFO/Fluorol 7GA/TiO_2_ NP OLEDs, at the maximum luminescence, are presented in Figure 4. Although all devices yielded broad EL spectra as observed in the PL spectra [8], detailed investigation revealed several remarkable dissimilarities. This observation was primarily due to different excitation sources. While both spectra indicated Förster an energy transfer mechanism, the use of electrical excitation source resulted in the creation of a carrier trapping center, as proved by the reduction in turn-on voltage.

Based on Figure 3, the PFO and Flourol 7GA HOMO barrier heights at the electrode (ITO) interface are 1.1 eV and 0.5 eV, respectively. Thus, due to lower barrier height, holes are more easily injected into Fluorol 7GA than into PFO. On the other hand, the incorporation of the TiO_2_ NPs into the PFO/Fluorol 7GA blends successfully reduced the LUMO barrier height at the electrode (Al) interface from 1.3 to 0.1 eV and thus improved electron injection. The accumulation of holes and electrons at Flourol 7GA and TiO_2_ NPs, respectively, had consequently enhanced the EL intensities.

The EL intensity recorded steady increments with TiO_2_ NP content, and reached a maximum emission intensity at 20–25 wt % TiO_2_ NPs before being dramatically reduced at a high concentration. The reduction of the EL intensity above 25 wt % can be attributed to the electrons tunneling through the emissive layer of PFO/Fluorol 7GA without recombining with the holes [11,20]. Moreover, the high content of the TiO_2_ NPs may create an imbalance between electrons and holes, which caused reduction in further charge transport.

It is interesting to note that the blue emission peaks commonly associated with the PFO (blue emission) improved upon additions of the TiO_2_ NPs with ratios up to 15 wt %. Low keto (fluorenone) defect content, which is associated with greater PFO resistance towards oxidation, was reported as the plausible reason for the improvement of blue emission. Meanwhile, the reduction in energy gap of PFO with increment TiO_2_ NPs [9] led to the strengthening of the condition of energy transfer from PFO to Fluorol 7GA and thus the enhancement of green to yellow emissions. Thus, the EL spectra recorded in the current work as well as PL spectra in the previous report [8] provided strong evidence that the presence of both TiO_2_ NPs and Fluorol 7GA in PFO enhanced the resistance of PFO towards oxidation and hence remarkably reduced the concentration of keto defects. However, as the TiO_2_ NP content exceeded 15 wt %, the blue emission was dramatically reduced with the 453 nm peak and completely disappeared. Although the content of keto defects may be further reduced at higher TiO_2_ concentrations, the continuous reduction in blue emission intensity is believed to be due to TiO_2_ NP agglomeration [11,21]. TiO_2_ nanoparticle agglomeration in related systems was evident indeed from FE-SEM images in our previous report on similar systems [10].

### 3.3. Luminance (cd/cm^2^) and Luminance Efficiency (cd/A)

**Figure 5:**
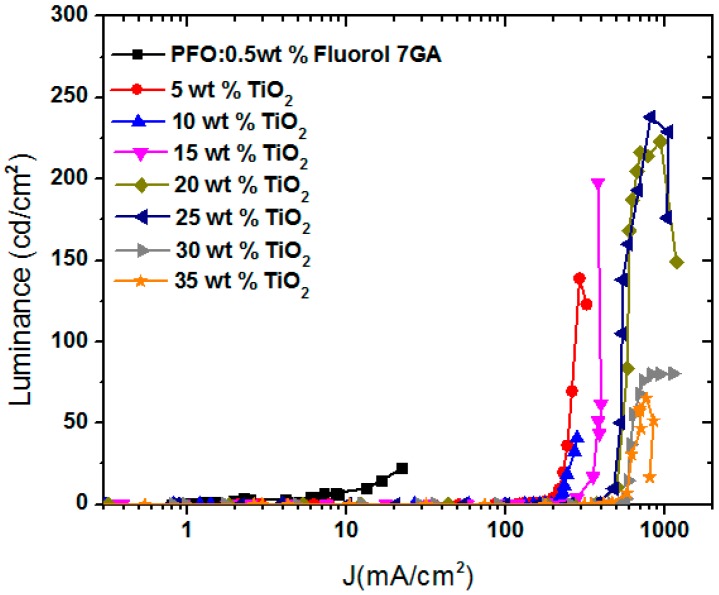
Luminance—current density characteristics for PFO/Fluorol 7GA with various weight ratios of TiO_2_ NPs.

As shown previously in Figure 3, upon addition of the TiO_2_ NPs into the PFO/Fluorol 7GA blend, the LUMO barrier height at the electrode (Al) interface decreased from 1.3 to 0.1 eV. As a result, the incorporation of the TiO_2_ NPs in the PFO/Fluorol 7GA blend improved electron injection from the cathode (Al); meanwhile, the Fluorol 7GA blocked these high electrons′ mobility. Consequently, the devices exhibited high luminance, as revealed in Figure 5.

The luminance of the PFO/Fluorol 7GA OLED device was improved upon increment TiO_2_ NPs with the optimal ratio of 20–25 wt %. At this ratio, the OLED displayed a maximum luminance of 238 cd/m^2^ at 20 V, corresponding to a current density of 813 mA/cm^2^ and luminance efficiency of 0.03 cd/A. However, further additions of TiO_2_ NPs resulted in the reduction of OLED luminance as shown in Table 1. This observation suggests that, as the electrons’ mobility was quite high with a greater amount of TiO_2_ NPs, the electrons managed to tunnel through the emissive layer without recombining with the holes, resulting in the reduction in luminance intensity. Similar observations has been reported for the systems of MEH-PPV/DNA/SWCNT and PF-oxe/ZnO [20,22].

In addition, the maximum luminance efficiency was for 15 wt % of TiO_2_ NPs at which the intensity of the blue EL emission (Figure 4) was the highest compared with other ratios. Above this critical ratio, a reduction in the blue EL emission was observed and thus the luminance efficiency declined, as demonstrated in Table 1. This observation may indicate that a high concentration of the TiO_2_ NPs has a negative impact on Förster energy transfer between PFO and Fluorol 7GA and thus reduce the green to yellow emission intensity. This was supported by the suppression of the Fluorol 7GA emission peaks when the TiO_2_ NP content exceeded 25 wt % [8].

### 3.4. Morphology of (PFO/Fluorol 7GA)/TiO_2_ Nanocomposite Films

**Figure 6:**
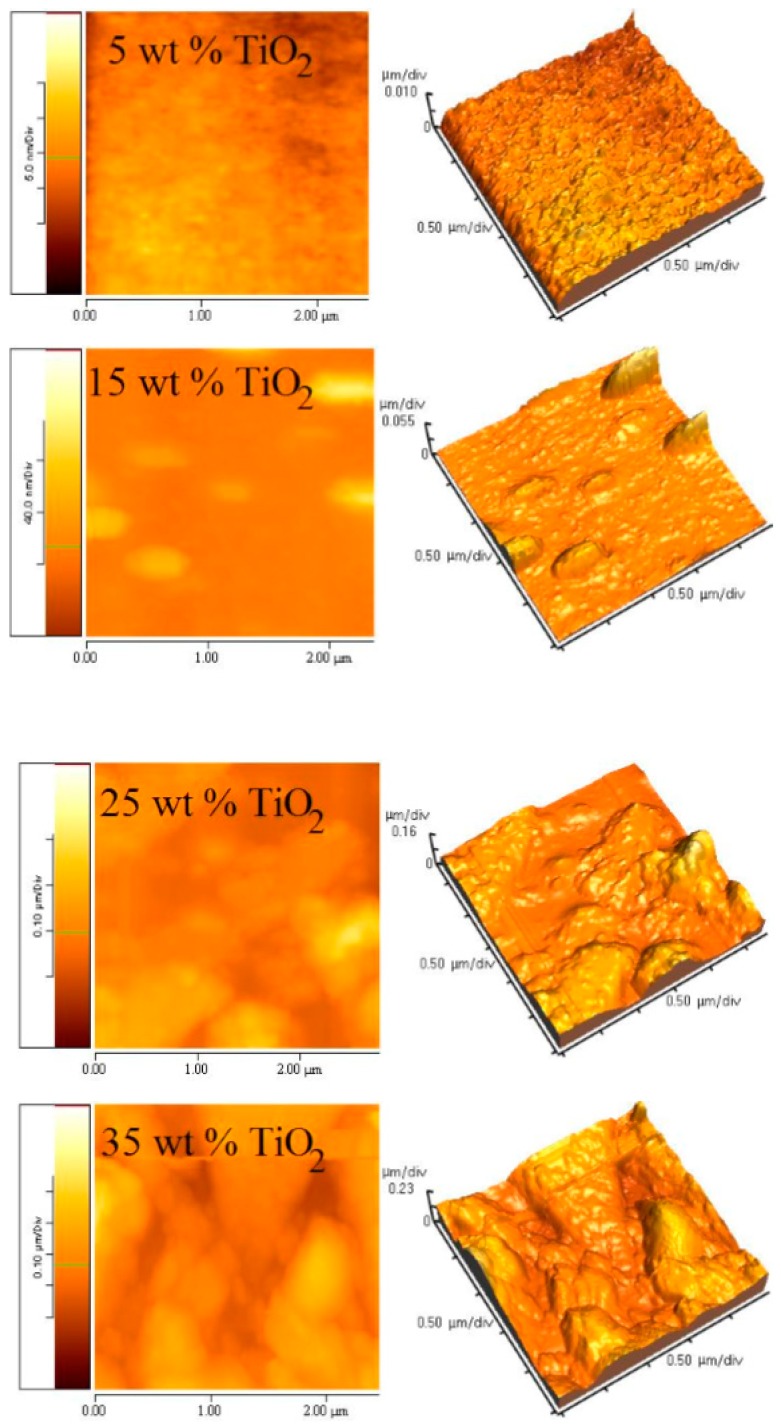
2D and 3D SPM images of PFO/0.5 wt % Fluorol 7GA blends with different content of TiO_2_ NPs, measured over an area of 2.5 × 2.5 μm^2^.

**Figure 7:**
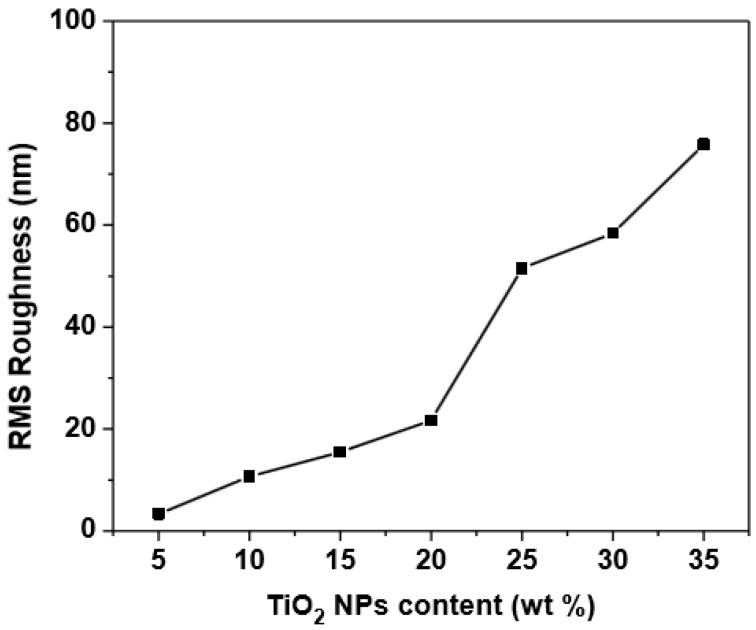
Root mean square (RMS) roughness for PFO/0.5 wt % Fluorol 7GA/TiO_2_ films as a function of TiO_2_ NP content.

The effect of TiO_2_ NPs on the surface morphology of PFO/Fluorol 7GA is shown in Figure 6. As the TiO_2_ NPs increased, embossment on the surface of the composite films was observed, as well as high surface roughness, as revealed in Figure 7. It can be found that the films roughness remarkably increased from 3.34 to 75.79 nm upon increments of the TiO_2_ NPs from 5 to 35 wt %. The PFO/Fluorol 7GA during deposition was damped in the TiO_2_ NPs in an effect identified as capillarity, which subsequently increased the surface roughness.

The surface morphology and roughness indicate aggregation as well as phase separation with a high amount of TiO_2_ NPs. Other studies have revealed that the larger contact area with cathodes corresponding to a rougher surface would increase a locally high electric field, resulting in more efficient electron injection and hence lower turn-on voltage [23,24].

The larger values of roughness (for 30 wt % and 35 wt % of TiO_2_) led to high aggregation and phase separation, which may cause short-circuit device due to high leakage current, and then reduced the luminance efficiency of the device, as illustrated in Table 1.

## 4. Conclusions

The solution blending method was successfully used to prepare (PFO/Fluorol 7GA)/TiO_2_ nanocomposites, which were used as an emissive layer in OLED devices. The decrease in turn-on voltage and opposite trend in the EL, luminance, and luminance efficiency of the device suggested an improvement in the device performance as TiO_2_ content was increased. The aggregation and phase separation at a high amount of TiO_2_ NPs led to efficient electron injection and hence lower turn-on voltage. However, further additions of TiO_2_ NPs (>25 wt %) resulted in the reduction of OLED performance in terms of EL, luminance, and luminance efficiency. The optimal ratio of TiO_2_ NPs was 20–25 wt % for EL and luminance, while it was 15 wt % for luminance efficiency. This work successfully demonstrated that the combination between the donor/acceptor (PFO/Fluorol 7GA) blending and incorporation of nanostructures (TiO_2_ NPs) into the conjugated polymer (PFO) was presented as a novel method to improve the performance of the PFO-based LED devices. The performance of these devices can be further improved by improving proceeding conditions and optimizing the structure of the devices.

## Figures and Tables

**Table 1:** Optoelectronic characteristics of PFO/0.5 wt % Fluorol 7GA/TiO_2_ light emitting diodes at various contents of TiO_2_ NPs.

Emissive layer	Max. luminance (cd/m^2^)	Luminance efficiency ^(a)^ (cd/A)	Turn-on voltage ^(b)^ (V)	Current density ^(a)^ (mA/cm^2^)
5 wt % TiO_2_	40.7 at 29 V	0.014	20	280.0
10 wt % TiO_2_	139 at 26 V	0.048	16.5	290.6
15 wt % TiO_2_	198 at 21 V	0.052	15	378.7
20 wt % TiO_2_	223 at 20 V	0.024	12	937.7
25 wt % TiO_2_	238 at 20 V	0.029	12.5	813.0
30 wt % TiO_2_	80.5 at 24 V	0.007	15	1124.1
35 wt % TiO_2_	65.3 at 19 V	0.009	12	764.2

^(a)^ At maximum luminance; ^(b)^ At luminance of 0.5 cd/m^2^ as standard.
